# Can urinary biomarkers predict acute kidney injury in newborns with critical congenital heart disease?

**DOI:** 10.3906/sag-2004-370

**Published:** 2021-02-26

**Authors:** Özgün UYGUR, Özge ALTUN KÖROĞLU, Ertürk LEVENT, Eser SÖZMEN, Fırat ERGİN, Yüksel ATAY, Mehmet YALAZ, Mete AKISÜ, Nilgün KÜLTÜRSAY

**Affiliations:** 1 Department of Pediatrics, Faculty of Medicine, Ege University İzmir Turkey; 2 Department of Biochemistry, Faculty of Medicine, Ege University, İzmir Turkey; 3 Department of Cardiovasculer Surgery, Faculty of Medicine, Ege University, İzmir Turkey

**Keywords:** Acute kidney injury, cardiovascular surgery, critical congenital heart disease, newborn, urinary biomarker

## Abstract

**Background/aim:**

Congenital heart disease (CHD) is the most common congenital malformation group and is the leading cause of newborn mortality in developed countries. Most of the infants with CHD develop preoperative or postoperative acute kidney injury (AKI). Acute kidney injury may develop before the serum creatinine rise and oliguria. Urinary biomarkers such as kidney injury molecule-1 (KIM-1), neutrophil gelatinase-associated lipocalin (NGAL), interleukin (IL)-18, and cystatin C may predict AKI in patients with critical CHD (CCHD) before the serum creatinine rise. In this study, we aimed to determine the AKI incidence among newborn patients with CCHD and investigate the predictivity of urinary biomarkers for AKI.

**Materials and methods:**

Newborns with a gestational age >34 weeks and birth weight >1500 g with a diagnosis of CCHD were enrolled in the study. Blood and urine samples were collected at birth, during the first 24–48 h, and in the preoperative and postoperative periods.

**Results:**

A total of 53 CCHD patients requiring surgery during the neonatal period were enrolled in the study. The 24–48 h KIM-1 levels of the cases with exitus were higher (P = 0.007). The 24–48 h cystatin C and preoperative NGAL levels were higher in patients with postoperative AKI (P = 0.02).

**Conclusion:**

In newborns with CCHD, high KIM-1 levels may predict mortality, whereas high cystatin C and preoperative NGAL levels may be indicative of AKI. These biomarkers deserve further investigation in larger study populations.

## 1. Introduction

Congenital heart disease (CHD) is the most common congenital malformation in the neonatal period and is a leading cause of neonatal mortality. Acute kidney injury (AKI) may develop preoperatively or postoperatively in neonatal CHD patients. In newborns, oliguria alone is not sufficiently sensitive to assess renal function and diagnose renal failure. Furthermore, AKI may develop without oliguria [1–3]. Therefore, better methods are needed for early and accurate diagnoses of AKI in newborns [4].

Although the data on the incidence of AKI in newborns is limited, the reported incidence in neonatal intensive care units (NICUs) is 8%. Most newborns with AKI are premature and/or critically ill newborns [5]^1^1Martin R, Stapleton FB (2015). Acute kidney injury (acute renal failure) in the newborn [online]. Website www.uptodate.com/contents/neonatal-acute-kidney-injury-pathogenesis-etiology-clinical-presentation-and-diagnosis [accessed 08/05/2015]. . Prerenal renal failure is the most common etiology (85%) in newborns and may result in intrinsic kidney failure if it is not treated promptly [6].

The criteria of neonatal RIFLE (risk, injury, failure, loss of function, end-stage kidney disease) and the Acute Kidney Injury Network (AKIN) have been established by modifying the pediatric RIFLE criteria defined in 2007 in order to diagnose AKI in newborns, and an attempt has been made to standardize the diagnostic criteria using these standards [1,5]. However, the criteria used to diagnose AKI in neonates hospitalized in NICUs are limited in their definition of kidney damage as they are based on the serum creatinine level and urine volume. Therefore, the neonatal-modified KDIGO (kidney disease: improving global outcomes) criteria based on serum creatinine rise and urine output, in addition to serum creatinine levels, is commonly used [7,8].

The use of new markers has been increasing due to the limitations described above. The ideal biomarker should be readily available in biological samples (e.g., urine), be sensitive enough to detect mild renal impairment, have a high predictive sensitivity for prognosis, and be specific to acute renal failure (ARF) and independent of glomerular filtration rate. Acute kidney injury develops in more than half of the patients with critical congenital heart disease (CCHD) in the postoperative period [9]. The limitations of serum creatinine include a lack of steady-state conditions in critically ill patients and inhibition of timely management. Early diagnosis and treatment will improve prognoses and help to create new types of therapeutic management [5].

To date, there have been a limited number of studies about urinary biomarkers, especially for predicting AKI in neonates. Some of the most promising urinary biomarkers for predicting AKI are cystatin C, IL-18, NGAL, and KIM-1. These studies of neonates have mostly centered on specific groups at risk for AKI such as very low birth weight infants, asphyxiated newborns, and newborns undergoing major cardiac surgery [5]. 

In the current study, we aimed to determine the AKI incidence in infants with CCHD and predict AKI before the serum creatinine rise by using urinary biomarkers. 

## 2. Materials and methods

Newborns with a gestational age >34 weeks, a birth weight >1500 g, and a diagnosis of CCHD were followed up in the NICU of the Ege University Faculty of Medicine and enrolled in the study. Informed consent was obtained from all parents of patients who were eligible for the study. Patients with major malformations other than cardiac disease; patients with renal anomalies, neonatal sepsis, or perinatal asphyxia; and cases in which family consent was not given were excluded. Ethics committee approval for the study was obtained from the Ege University Medical Faculty Local Ethics Committee (date: 14.08.2013; no.: 13-4.1/8).

General supportive care was provided to all patients according to the NICU protocol. Demographic characteristics for all newborns were recorded. Blood samples (2 mL) were collected from the cord, and urine samples (2 mL) were obtained from the urinary bag during the first 24 h. In this period, while urine was collected to determine the possibility of antenatal renal damage, blood was collected to calculate the creatinine clearance to account for the maternal effect on creatinine during the first 24 h of life. Further blood and urine samples were collected in the first 24–48 h of life, preoperatively, and 24–48 h postoperatively. After 24 h, the blood samples were collected from the umbilical or central venous catheter, and the urine samples were collected from the urine catheter.

All urinary biomarkers were obtained from Shangai Sunred Biological Technology (China) [catalog numbers: 201-12-1720 (NGAL), 201-12-1100 (KIM-1), 201-12-1105 (cystatin C), and 201-12-0148 (IL-18)] and analyzed by ELISA method. Serum creatinine measurements were performed spectrophotometrically by the Jaffe method. Creatinine clearance was calculated using serum creatinine level and height (centimeters). Neonatal KDIGO staging was performed to diagnose and evaluate ARF. Neonatal KDIGO staging is as follows: stage 0, no change in serum creatinine (SCr) or a rise of 0.3 mg/dL with urine output >0.5 mL/kg/h; stage 1, SCr rise ≥0.3 mg/dL within 48 h or SCr rise ≥1.5–1.9 × reference SCr within 7 days with urine output <0.5 mL/kg/h for 6 to 12 h; stage 2, SCr rise ≥2.0–2.9 × reference SCr with urine output <0.5 mL/kg/h for ≥12 h; stage 3, SCr rise × 3 reference SCr or SCr level ≥ 2.5 mg/dL or receipt of dialysis with urine output <0.3 mL/kg/h for ≥24 h; stage 4, anuria for ≥12 h. 

In our study, group 1 consisted of patients without acute renal damage, and group 2 was comprised of patients with acute renal damage developed at any time during the study period. In patients undergoing surgery, the presence of cardiopulmonary bypass, hypothermia, and extracorporeal membrane oxygenation (ECMO), as well as cross clamping status and duration were recorded. Neutrophil gelatinase-associated lipocalin (NGAL), kidney injury molecule (KIM-1), cystatin C, and interleukin (IL)-18 levels were examined in the urine. These biomarkers were examined for every CCHD patient during four time periods: the first 24 h of life, the first 24–48 h of life, preoperatively, and 24–48 h after the operation. The levels of urinary biomarkers of all patients from each of these time periods were examined and compared according to AKI development at any stage of the study.

### 2.1. Statistical analysis

SPSS version 21.0 for Windows (IBM Corp., Armonk, NY, USA) was used to evaluate the data. Statistical analysis was performed using the SPSS for Windows statistical package. The χ2 test was used to compare categorical variables between groups. The Kolmogorov–Smirnov test was used to evaluate the normal distribution assumption for numerical variables. The difference between two groups was examined by independent samples t-test for normally distributed variables and the Mann–Whitney U-test for nonnormally distributed variables. Differences among more than two groups were examined by ANOVA with repeated measures. Mauchly’s test of sphericity and Greenhouse–Geisser corrections were applied to test the sphericity assumption before ANOVA, with repeated measures. A paired t-test with the Bonferroni correction was used for multiple comparisons between tests. In addition, a paired t-test was used to compare the arithmetic averages of two independent groups. A P value of <0.05 was considered statistically significant.

## 3. Results

The study included a total of 53 newborns requiring major cardiac surgery due to CCHD. Demographic variables and the cardiac diagnoses of the patients are summarized in Table 1. 

**Table 1:** Demographic variables of the patients.

	n = 53
Gestational age, weeks mean±SD (min-max)	38.0 ± 1.7 (34-41)
Birth weight, grams mean ± SD (min-max)	2932 ± 654 (1510-4000)
Term, n (%)Late preterm, n (%)	43 (81.2)10 (18.8)
Sex, male (%)	33 (62.3)
Cardiac diagnosis, n (%) Aort coarctation Transposition of the great arteries Pulmonary atresia Tricuspit atresia Hypoplastic left heart syndrome Fallot tetralogy Aort coarctation with transposition of the great arteries Truncus arteriosus Double outlet right ventricle with atrioventricular septal defect	14 (26.4) 12 (22.6) 7 (13.2) 7 (13.2) 6 (11.3) 3 (5.7) 2 (3.8) 1 (1.9) 1 (1.9)

In our study group, 69.8% of the patients (n = 37) developed AKI during the study period. In 18 patients (34%), AKI developed only in the postoperative period. Among all patients, only one (1.9%) required peritoneal dialysis during the postoperative period.

The urine specimens and creatinine clearance from 4 different time periods (the first 24 h of life, the first 24–48 h, preoperative, and 24–48 h postoperative) for each patient with CCHD were compared. The urinary biomarker levels are shown in Table 2. The mean values of biomarkers were also compared to each other for every two time periods; there was a significant difference between the 24–48 h and preoperative values of cystatin C (P < 0.05). In addition, the difference between the 24–48 h and preoperative values of IL-18 was significant (P < 0.05). In urinary NGAL values, a significant difference was found between time periods: P < 0.05 for cord vs. preoperative values and P < 0.05 for 24–48 h vs. preoperative values. A significant difference was detected between time periods for urinary KIM-1 values at 24–48 h vs. preoperative values (P < 0.05). On the other hand, urinary KIM-1 levels at 24–48 h of life were higher in patients who were lost during the postoperative period (P < 0.05). 

**Table 2:** Levels of urine examinations of all patients according to time periods.

	First 24 h	24-48th h	Preoperative	Postoperative	P*
Cystatin C (ng/mL)	18.19 + 1.06a	19.12 ± 0.96a,b	18.20 ± 1.23a,b	20.97 ± 0.79a	0.81
IL-18 (ng/mL)	19.40 + 0.86a	18.91 ± 0.73a,b	17.19 ± 0.66a,b	18.48 ± 0.78a	0.73
NGAL (ng/mL)	314.39 ± 54.20a,b	249.47 ± 45.04a,b	223.35 ± 32.59a,b	303.41 ± 40.44a	0.79
KIM-1 (ng/mL)	1.46 ± 0.14a	1.45 ± 0.12a,b	1.48 ± 0.07a,b	1.62 ± 0.12a	0.69

* ANOVA with repeated measures is used for the comparison of each biomarker according to time periods. Paired t-test with Bonferroni correction was used for multiple comparisons between tests (a > 0.05; b < 0.05).

The most remarkable point was the cystatin C levels at 24–48 h of life; the NGAL levels in the preoperative period were significantly higher in patients who developed AKI in the postoperative period (P = 0.02). 

The levels of four urinary biomarkers were examined in all time periods for each patient by grouping the patients according to presence or absence of AKI. These results are summarized in Table 3. Urinary IL-18 at 24–48 h of life was significantly higher in patients developing AKI; however, no predictive value was detected for postoperative development of AKI. ECMO was performed on three patients during the study; only one patient developed AKI, and the damage started preoperatively. During the study, 13 patients underwent cardiopulmonary bypass and of these patients, seven developed AKI (only one case emerged preoperatively). 

**Table 3:** The levels of urinary biomarkers according to AKI at all time periods.

	First 24 h(AKI n = 0)			24-48th h of life*(AKI n = 15)			Preoperative(AKI n = 14)			Postoperative(AKI n = 27)		
	Group 1(n = 16)	Group 2(n = 37)	P	Group 1(n = 16)	Group 2(n = 37)	P	Group 1(n = 16)	Group 2(n = 37)	P	Group 1(n = 16)	Group 2(n = 37)	P
Cystatin C (ng/ml) mean±SD	16.71 *(16.3-31.01)	16.16 *(11.95-28.07)	0.24	13.89 ± 4.46	20.39 ± 4.29	0.06	15.76 ± 3.12	17.82 ± 9.17	0.59	20.17 ± 2.46	22.12 ± 5.44	0.85
IL-18 (ng/ml) mean ± SD	19.54 ± 5.90	19.18 ± 4.25	0.93	15.01 ± 3.60	18.98 ± 4.60	0.03	16.34 ± 2.60	18.01 ± 4.88	0.11	19.45 ± 4.34	18.14 ± 6.03	0.20
NGAL (ng/ml)*Median (min-max)	382 (28-933)	110.5(21-621)	0.37	60.5(19-417)	65(15-846)	0.80	80(19-417)	57(11-552)	0.26	34.5(7-452)	286 (12-785)	0.34
KIM-1 (ng/ml) mean ± SD	1.83 *(0.36-2.60)	1.63 *(0.08-2.16)	0.24	1.54 *(0.06-1.81)	1.69 *(0.13-2.02)	0.21	1.36 ± 0.70	1.36 ± 0.51	0.71	1.63 *(1.13-2.09)	1.83 *(0.32-3.42)	0.96
Serum creatinine (mg/dl) mean ± SD	0.78 ± 0.30	0.74 ± 0.23	0.60	0.67 ± 0.36	0.89 ± 0.33	0.06	0.60 ± 0.31	0.67 ± 0.37	0.04	0.55 ± 0.32	0.77 ± 0.40	0.006
Creatinine clirence mean ± SD	32.67 ± 15.18	32.73 ± 12.34	0.92	47.54 ± 36.06	27.55 ± 10.8	0.005	50.65 ± 35.22	44.55 ± 29.56	0.02	63.0 ± 46.97	37.71 ± 24.22	0.01

*Values are given as median (min-max).P values are defined using sample t-test and Mann-

## 4. Discussion

Acute renal injury is a frequent clinical complication observed during the preoperative or postoperative period in newborns with CCHD. Among the patients in our study, 69.8% developed AKI. Kumar et al. [10] reported an ARF incidence of 8% in a postoperative period of 72 h among infants <2 months old. In a study by Alabbas et al. [11], AKI was detected in 62% of the cardiac patients <28 days old, which is similar to our study. Acute kidney injury is also associated with prolonged hospitalization periods and an increase in mortality. Similarly, Blinder et al. [9] reported that 225 (52%) of 430 patients younger than 90 days old developed AKI in the postoperative period. Although there are various reports about AKI incidence, our results seem to be compatible with previous reports. In our study, serum creatinine levels and creatinine clearance were only significantly higher in the postoperative samples of patients with AKI. These results highlight the importance of further studies of urinary biomarkers in order to detect AKI at earlier stages. 

The serum cystatin C level in neonates is one of the promising parameters in the diagnosis and follow-up of AKI. Cystatin levels are not affected by gender, size, or gestational week and are also independent of body mass and serum bilirubin levels. Furthermore, fetal cystatin C levels are not affected by maternal cystatin C levels [12]. In our study, cystatin C values in urine were high in the CCHD group, and although it was not significant, an increase in cystatin C was detected in the postoperative period. Urine cystatin C levels were significantly higher at 24–48 h of life in patients who went on to develop AKI after the operation. A total of 374 children were included in a study by Krawczeski et al. [13], and NGAL levels of urine and serum were examined from 35 newborns ˂ 30 days of life after cardiopulmonary bypass. In this study, serum cystatin C concentrations increased significantly in AKI patients 12 h after cardiopulmonary bypass (P < 0.0001) and remained elevated at 24 h (P < 0.0001) [13]. Additionally, Zhang et al. [14] reported that cystatin C is an excellent predictor of AKI. 

The IL-18 level in urine is considered an early noninvasive biomarker for AKI [15]. Urinary NGAL and IL-18 in children undergoing cardiac surgery have recently been shown to be biomarkers revealing AKI. In our study, urinary IL-18 levels at 24–48 h of life were significantly higher in patients who went on to develop AKI during any time period; however, IL-18 could not predict the inception of AKI in the postoperative period. Li et al. [16] used new urinary biomarkers to detect AKI in 62 critically ill neonates (8 with neonatal encephalopathy), and IL-18 levels in urine were significantly higher in critically ill newborns with nonseptic AKI compared to the control group [16]. 

Neutrophil gelatinase-associated lipocalin (NGAL) is regulated by mRNA induction in cases of infection, cancer, and renal tubular damage. In cases of renal epithelial damage, serum NGAL levels increase 7-16–fold and urinary NGAL levels increase 25-100–fold. In the pediatric and adult age groups, NGAL levels increase significantly in patients with AKI, and this increase begins 24–48 h before the elevation of serum creatinine [17]. In our study, NGAL levels were lower in the urine during the preoperative period and increased significantly in the postoperative period. Oncel et al. [18] reported elevated urinary NGAL levels during the first day among patients with perinatal asphyxia. In the study by Tanigasalam et al. [19], among 120 neonates exposed to perinatal asphyxia, 55 (46%) developed AKI. According to this study, a NGAL level of 86.82 ng/mL had a sensitivity of 87% and a specificity of 87.7% to predict AKI. 

The KIM-1 molecule is an indicator that is being studied as an early-period marker of AKI in adults. The KIM-1 levels in urine can distinguish ischemic kidney damage according to prerenal azotemia and chronic kidney disease [20]. Han et al. [21] stated that among 40 patients who underwent cardiac surgery, urinary KIM-1 values were high in 20 patients who were developing AKI. In our study, the mean KIM-1 levels were higher in the postoperative period compared to the other periods, and the 24–48 h KIM-1 levels of the patients who died in this group were significantly higher. 

 In conclusion, AKI is commonly seen in NICUs and results in undesired effects on morbidity and mortality. Studies of urinary biomarkers are limited in neonates with CCHD, and to date there is no internationally accepted biomarker for AKI. According to our results, in newborns with CCHD, high KIM-1 levels may predict mortality, whereas high cystatin C (during the first 24–48 h of life) and preoperative NGAL levels may be indicative of the postoperative development of AKI. These biomarkers deserve further investigation in larger study populations. 

## Funding source

This study was funded by a local scientific study project group (BAP-2014-TIP-038).

## Financial disclosure

The authors have no financial relationships relevant to this article to disclose.

## Informed consent

The study protocol received institutional review board approval (Ege University Medical Faculty Local Ethics Committee; date: 14.08.2013/no.: 13-4.1/8) and all participants provided informed consent in the format required by the relevant authorities.
